# Cerebellar contribution to sensorimotor adaptation deficits in humans with spinal cord injury

**DOI:** 10.1038/s41598-020-77543-8

**Published:** 2021-01-28

**Authors:** Yuming Lei, Monica A. Perez

**Affiliations:** 1 Department of Health and Kinesiology, Texas A&M University, College Station, TX 77845 USA; 2 Shirley Ryan Ability Lab, Northwestern University, Edward Hines, Jr. VA Hospital, Northwestern University, Chicago, IL 60611 USA; 3 The Miami Project to Cure Paralysis, Bruce W. Carter Department of Veterans Affairs Medical Center, University of Miami, Miami, FL 33136 USA

**Keywords:** Motor control, Spinal cord

## Abstract

Humans with spinal cord injury (SCI) show deficits in associating motor commands and sensory feedback. Do these deficits affect their ability to adapt movements to new demands? To address this question, we used a robotic exoskeleton to examine learning of a sensorimotor adaptation task during reaching movements by distorting the relationship between hand movement and visual feedback in 22 individuals with chronic incomplete cervical SCI and 22 age-matched control subjects. We found that SCI individuals showed a reduced ability to learn from movement errors compared with control subjects. Sensorimotor areas in anterior and posterior cerebellar lobules contribute to learning of movement errors in intact humans. Structural brain imaging showed that sensorimotor areas in the cerebellum, including lobules I–VI, were reduced in size in SCI compared with control subjects and cerebellar atrophy increased with increasing time post injury. Notably, the degree of spared tissue in the cerebellum was positively correlated with learning rates, indicating participants with lesser atrophy showed higher learning rates. These results suggest that the reduced ability to learn from movement errors during reaching movements in humans with SCI involves abnormalities in the spinocerebellar structures. We argue that this information might help in the rehabilitation of people with SCI.

## Introduction

Sensorimotor adaptation learning is important for skill acquisition and rehabilitation^1,2^. This learning depends on the ability to integrate predicted sensory consequences of motor actions with sensory feedback^3,4^ and it is affected after brain damage^5,6^. Humans with spinal cord injury (SCI) show deficits in sensorimotor function^7^. Indeed, these individuals present difficulties during tasks requiring visuo-motor integration^8–10^ and in their ability to form body representations^11,12^. However, the extent to which humans with incomplete cervical SCI can learn to adapt their arm movements to novel perturbations remains unknown.

Adaptation learning of arm movements has been widely studied by distorting the relationship between hand movement and visual feedback, which results in movement errors^13^. Electrophysiological studies in non-humans primates showed that adaptation learning requires the functional integrity of the cerebellum^14–16^. In agreement, neuroimaging studies in humans showed that cerebellar lobules I–VI contribute to adaptation learning during reaching movements^6,17,18^. The integrity of the cerebellum is disrupted after SCI. Animal models of SCI showed that cerebellar circuits undergo changes after injury due to a loss of spinocerebellar neurons^19^, which convey proprioceptive and cutaneous information from the periphery to the cerebellum^20^ and efferent copies for motor prediction^21–23^. Purkinje cells drive cerebellum-dependent motor learning^24,25^, and their number^26^ and synaptic contacts^19^ decreases after SCI. In humans, axons carrying proprioceptive information from the cervical spinal cord terminate in cerebellar lobules^27,28^. Proprioceptive signals, which are important for learning from movement errors^29,30^, are often impaired after SCI^7^ and individuals with SCI show evidence of cerebellar atrophy^31,32^. We hypothesized that people with SCI exhibit deficits in sensorimotor adaptation learning compared with uninjured controls, likely associated with abnormalities in the cerebellum.

To test our hypothesis, we used a robotic exoskeleton to examine adaptation learning during a visuo-motor rotation task in humans with and without chronic incomplete cervical SCI. Brain imaging was used to examine the relationship between cerebellar structure and sensorimotor adaptation learning. We found that humans with SCI show deficits in learning from movement errors compared with uninjured controls and that cerebellar atrophy correlated with abnormalities in adaptation learning. These findings provide evidence for a mechanism to explain difficulties in learning visuo-motor interactions after SCI and suggest that this learning might be important for rehabilitation of arm movements.

## Results

### Visuo-motor rotation task

**Figure 1:**
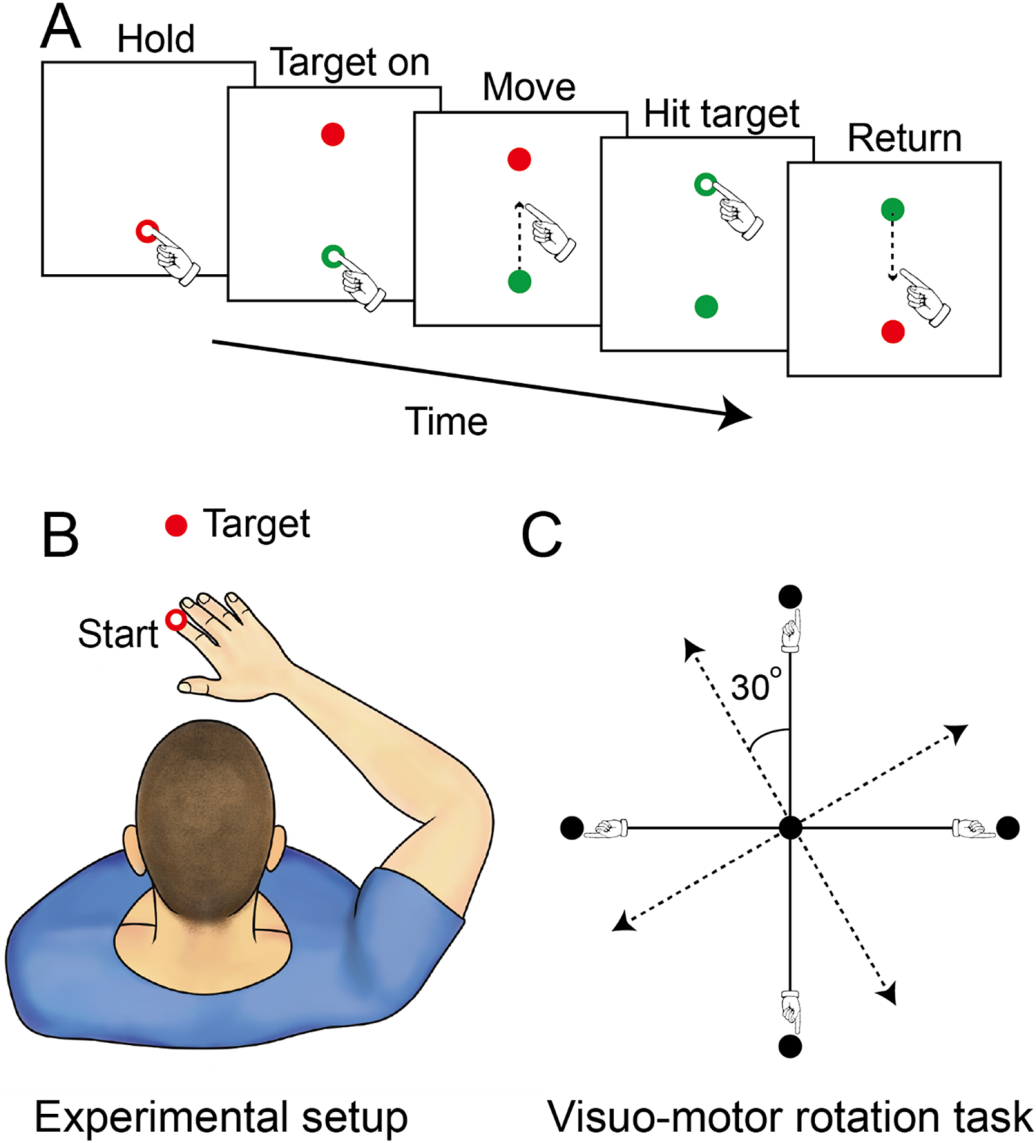
Experimental setup. (**A**) Each trial began when subjects centered the cursor (white circle, 0.5 cm in diameter) on the constant start circle (red circle, 1 cm in diameter). The target was randomly displayed at one of four locations 10 cm apart from the start circle. Subjects were instructed to reach in a straight and fast motion toward the target. (**B**) Subjects were seated upright on the KINARM chair. Visual targets and hand-aligned feedback were projected on a horizontal display via a virtual reality system. (**C**) Experimental setup for the visuo-motor rotation task. Visual display of the cursor representing fingertip location was rotated 30° counterclockwise.

**Figure 2:**
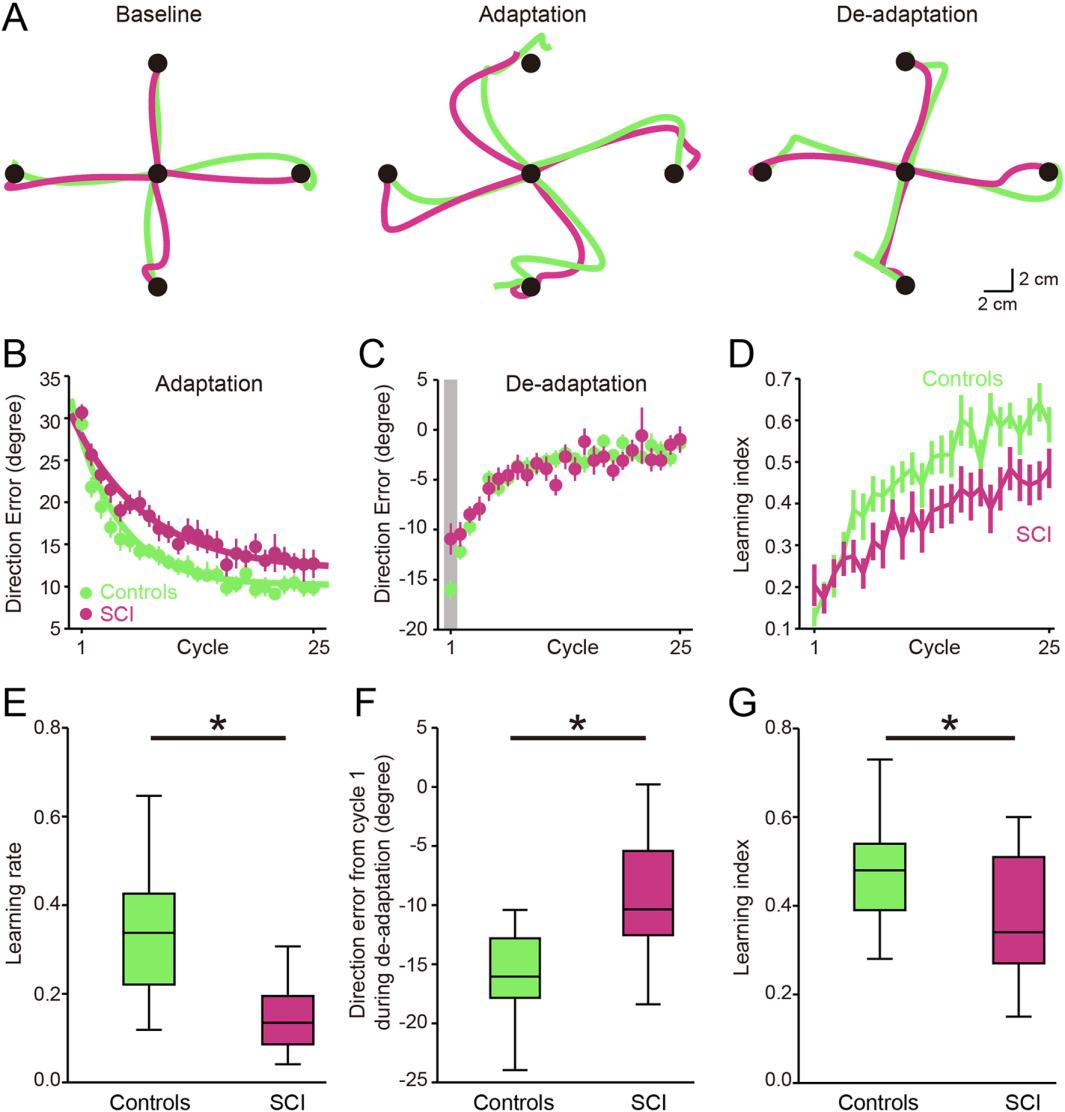
Visuo-motor rotation task. (**A**) Hand-paths of a representative control (green) and SCI (magenta) subject. Hand-paths in the left panel represent the last four consecutive trials observed during the baseline session; hand-paths in middle and right panels represent the first four consecutive trials observed during the adaptation and de-adaptation sessions, respectively. (**B**, **C**), Group data showing changes in direction errors at peak tangential arm velocity during the adaptation (**B**) and de-adaptation (**C**) sessions. Each data point shown on the x-axis represents the average of four consecutive trials (one cycle). The 25 cycles cover all the 100 trials. (**D**) Learning index for control and SCI subjects. Each learning index is composed of four consecutive trials (one cycle). The 25 cycles cover all the 100 trials. (**E**–**G**) Box-plot group data showing learning rate of errors during the adaptation session (**E**), direction errors from the first cycle during the de-adaptation session (**F**), and mean learning index during the adaptation session (**G**). Error bars indicate SDs. *p < 0.05.

In all experiments, participants were instructed to make goal-directed reaching movements from a start position to an end target. At the start of each trial, the visual display consisted of a 1 cm diameter start circle (red circle; Fig. 1A,B) that maintained the same limb configuration across participants. The distance between the start circle and 4 targets presented in a pseudorandom sequence was 10 cm. Subjects were instructed to reach in a straight and fast motion towards each target. After reaching the target, the subjects were instructed to bring the hand back to the start circle. Subjects performed three consecutive sessions: baseline (80 trials), adaptation (100 trials), and de-adaptation (100 trials). Figure 2A illustrates the hand-paths of a representative control (green) and SCI (magenta) subject. Note that their hand-paths were largely curved in the first of four consecutive trials (middle panel) during the adaptation session. After removing visual rotation perturbation during the de-adaptation session (right panel), the hand-paths were curved in the opposite direction, indicating after-effects, but to a lesser extent in SCI compared with control subjects.

Repeated measures ANOVA showed an effect of GROUP (F_(1, 42)_ = 13.3, p = 0.001) and CYCLE (F_(2, 84)_ = 340.2, p < 0.001) on direction error. We found that direction errors were larger at the beginning of the adaptation and progressively decreased throughout the session in both groups but to a lesser extent in SCI compared with control subjects (Fig. 2B). After-effects were present when the visual rotation was removed during the de-adaptation session and decreased with subsequent reaching movements in both groups (Fig. 2C). Repeated measures ANOVA also showed an effect of GROUP (F_(1, 42)_ = 11.8, p = 0.004) and CYCLE (F_(2, 84)_ = 362.3, p < 0.001) on the learning index. The learning index progressive increased throughout the session but to a lesser extent in SCI compared with control subjects (Fig. 2D). Consistently, we found that learning rate of errors was higher in controls compared with SCI subjects (controls = 0.34 ± 0.16, SCI = 0.16 ± 0.13, p = 0.001; Fig. 2E). Direction errors from the first cycle during de-adaptation were larger in controls compared with SCI subjects (controls = − 15.6 ± 4.1°, SCI = − 10.1 ± 7.8°, p = 0.01; Fig. 2F). SCI subjects showed lower mean learning index compared with control subjects during the adaptation session (controls = 0.47 ± 0.12, SCI = 0.36 ± 0.16; p = 0.01; Fig. 2G). During the adaptation session, SCI subjects (0.76 ± 0.08 s) showed an increase in arm deceleration compared with control subjects (0.62 ± 0.07 s, p = 0.003). The increase in arm deceleration for SCI subjects is expected given that this phase of the movement is executed mainly online and largely relies on feedback control. No differences were found in arm acceleration between SCI (0.26 ± 0.04 s) and control subjects (0.24 ± 0.06 s, p = 0.2), possibly because we asked participants to complete ballistic reaching movements. Note that peak arm velocity (controls = 0.33 ± 0.06 m/s, SCI = 0.31 ± 0.07 m/s; p = 0.6), reaction time (controls = 0.42 ± 0.05 s, SCI = 0.43 ± 0.06 s; p = 0.5), and direction error (controls = 6.47 ± 1.35°, SCI = 7.86 ± 2.65°; p = 0.1) were similar between SCI and control subjects at baseline, suggesting that differences observed in visuomotor adaptations across groups are related to the inability to learn via error information and feedback.

**Figure 3:**
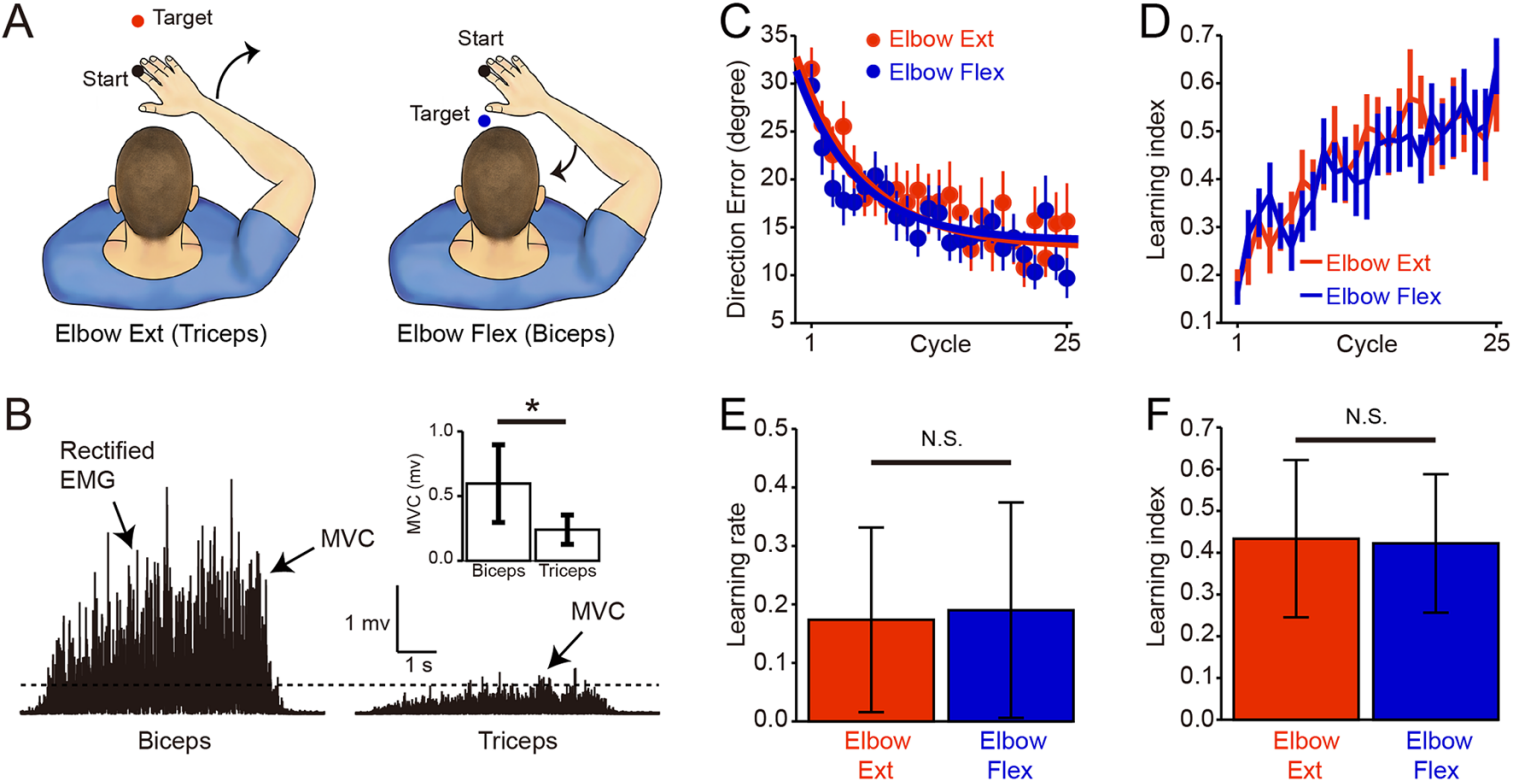
Muscle weakness and motor adaptation learning. (**A**) Subjects reached to the target required elbow extension (Elbow Ext = target positioned in the midline in front of the start circle) or elbow flexion (Elbow Flex = target positioned in the midline behind the start circle). (**B**) Rectified EMG activity in the biceps and triceps brachii in a representative SCI subject when the subject performed maximum voluntary contraction (MVC). Note that SCI subjects showed higher MVC values quantified by EMG signals in biceps compared with triceps brachii. (**C**, **D**) Group data showing changes in direction errors at peak tangential arm velocity during reaching movements towards a target where the reaching movement required elbow extension (red) or flexion (blue). (**E**, **F**) Group data showing learning rate (**E**) and mean learning index (**F**) during elbow extension and elbow flexion. Error bars indicate SDs. *p < 0.05.

We examined the effect of muscle weakness on sensorimotor adaptation learning by comparing the rate and magnitude of learning during reaching movements towards a target where the reaching movement required elbow extension or flexion (Fig. 3A). Repeated measures ANOVA showed an effect of CYCLE (F_(2, 36)_ = 85.8, p < 0.001), but not TARGET (F_(1,18)_ = 1.4, p = 0.3; Fig. 3C) on direction error. Repeated measures ANOVA also showed an effect of CYCLE (F_(2, 36)_ = 50.0, p < 0.001), but not TARGET (F_(1,18)_ = 0.02, p = 0.9; Fig. 3D) on learning index. No changes were observed in learning rate (elbow extension = 0.17 ± 0.16, elbow flexion = 0.19 ± 0.18; p = 0.4; Fig. 3E) and mean learning index (elbow extension = 0.43 ± 0.19, elbow flexion = 0.42 ± 0.17; p = 0.8; Fig. 3F) during elbow extension and elbow flexion. Since the handedness of our SCI subjects is affected by the injury and distinct control mechanisms contribute to the dominant and non-dominant arm^33,34^, we examined the contribution of handedness of our SCI subjects to our effects. The dominant arm was detected as the “more affected arm” in 13/22 SCI subjects after the injury. These SCI subjects also showed lower learning rate (controls = 0.34 ± 0.16, SCI = 0.18 ± 0.16; p = 0.01) and mean learning index (controls = 0.47 ± 0.12, SCI = 0.37 ± 0.20; p = 0.02) compared with controls.

### Upper extremity position sense and clinical assessment

Quantitative assessment of position sense of the upper extremity was conducted using the arm-position matching task^35^. We found that position accuracy errors were higher in SCI compared with control subjects (SCI = 7.1 ± 3.1 cm, controls = 3.1 ± 0.8 cm; p < 0.001). Position accuracy errors were not correlated with motor learning rates in SCI subjects (r = 0.23, p = 0.39). Clinical assessment of arm/hand motor function was conducted using the Jebsen Taylor Test^36^. We found that the time to complete the test increased in SCI compared with control subjects (controls = 14.7 ± 8.8 s, SCI = 4.7 ± 1.0 s; p < 0.001). The time to complete the JTT test was not correlated with motor learning rates in SCI subjects (r = 0.12, p = 0.51). These results indicated that adaptation learning deficits in SCI subjects were less likely related to impairments in proprioception and motor function.

### Structural MRI

**Figure 4:**
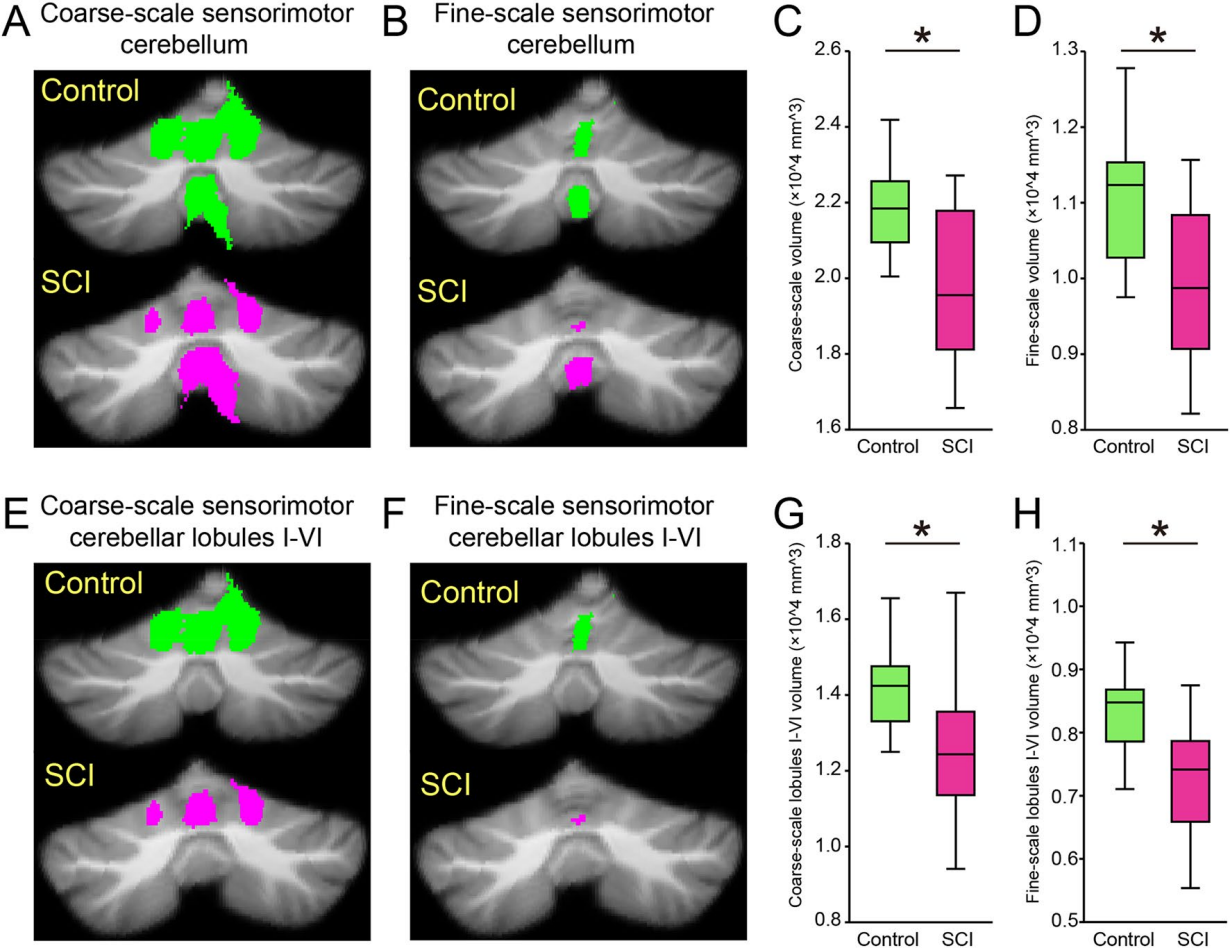
Structural MRI. (**A**, **B**), The coarse- and fine-scale sensorimotor cerebellum of a representative control (green) and SCI (magenta) subject. Note that the volume of coarse- and fine-scale sensorimotor cerebellum was smaller in SCI compared with control subjects. (**C**, **D**) Box-plot group data for the volume of coarse- and fine-scale sensorimotor cerebellum. (**E**, **F**) coarse- and fine-scale sensorimotor region in anterior and posterior cerebellar lobules identified in a representative SCI and control subject. Note that the volume of sensorimotor cerebellar lobules I–VI was also smaller in the SCI compared with the control subjects. (**G**, **H**) Box-plot group data for the volume of coarse- and fine-scale sensorimotor region in anterior and posterior cerebellar lobules I–VI. Error bars indicate SDs. *p < 0.05.

Figure 4A illustrates the coarse-scale sensorimotor cerebellum of a representative control (green) and SCI (magenta) subject. Note that the volume of the sensorimotor cerebellum was smaller in SCI compared with the control subject. In all subjects, the volume of the coarse-scale sensorimotor cerebellum decreased in SCI compared with control subjects (controls = 21,977.1 ± 1511.6 mm^3^, SCI = 19,763.4 ± 2067.8 mm^3^, p = 0.002; Fig. 4C). We also refined our assessment by focusing only on the sensorimotor cerebellar regions into hand and foot representations. In agreement, we found that the volume of fine-scale sensorimotor cerebellum was smaller in SCI compared with control subjects (controls = 11,039.6 ± 890.0 ${mm}^{3}$, SCI = 9810.9 ± 1096.9 ${mm}^{3}$, p = 0.002; Fig. 4B and D). Neuroimaging studies in humans showed that lobules I–VI in the anterior and posterior cerebellum are important for sensorimotor adaptation learning^5,6,18^. Figure 4E and F illustrate examples of coarse- and fine-scale sensorimotor regions in anterior and posterior cerebellar lobules identified in a representative SCI and control subject. Note that the volume of sensorimotor cerebellar lobules I–VI was smaller in the SCI compared with the control subjects. In all subjects, sensorimotor areas in cerebellar lobules I–VI decreased in SCI compared with control subjects (coarse-scale: controls = 14,260.8 ± 1361.0 ${mm}^{3}$, SCI = 12,428.3 ± 1928.4 ${mm}^{3}$, p = 0.004, Fig. 4G; fine-scale: controls = 8335.0 ± 847.3 ${mm}^{3}$, SCI = 7270.4 ± 1169.4 ${mm}^{3}$, p = 0.006, Fig. 4H). We found no differences in adaptation rate (C5–C6 = 0.158 ± 0.177, C2–C4 = 0.165 ± 0.106, p = 0.8), the amount of cerebellar atrophy (C5–C6 = 19,610.0 ± 2088.8 ${mm}^{3}$, C2–C4 = 19,993.5 ± 1651.1 ${mm}^{3}$, p = 0.6), and the time post-injury (C5–C6 = 9 ± 5 yrs, C2–C4 = 8 ± 4, p = 0.4) between participants with higher and lower cervical injuries.

### Correlations

**Figure 5:**
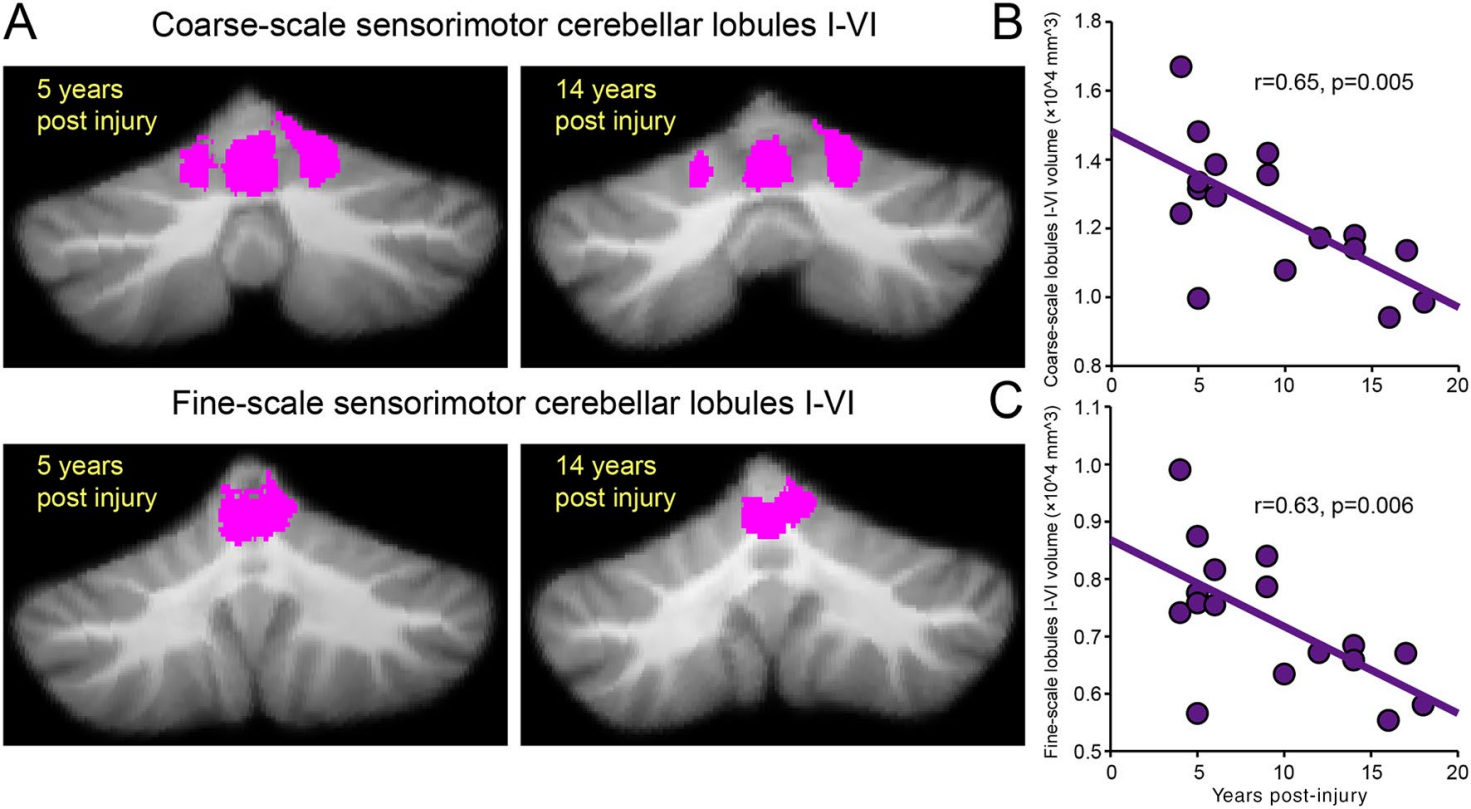
Cerebellar changes and time post-injury. (**A**) Coarse- and fine-scale sensorimotor regions in anterior and posterior cerebellar lobules identified in SCI subjects (SCI = 17) with 5 years post-injury (left column) and 14 years post-injury (right column). (**B**, **C**) Graphs show a correlation between the time post-injury and the volume of the sensorimotor cerebellum in SCI subjects. Note that SCI subjects with increasing time post-injury were those with smaller volume in the coarse- and fine-scale sensorimotor cerebellar lobules I–VI. *p < 0.05.

**Figure 6:**
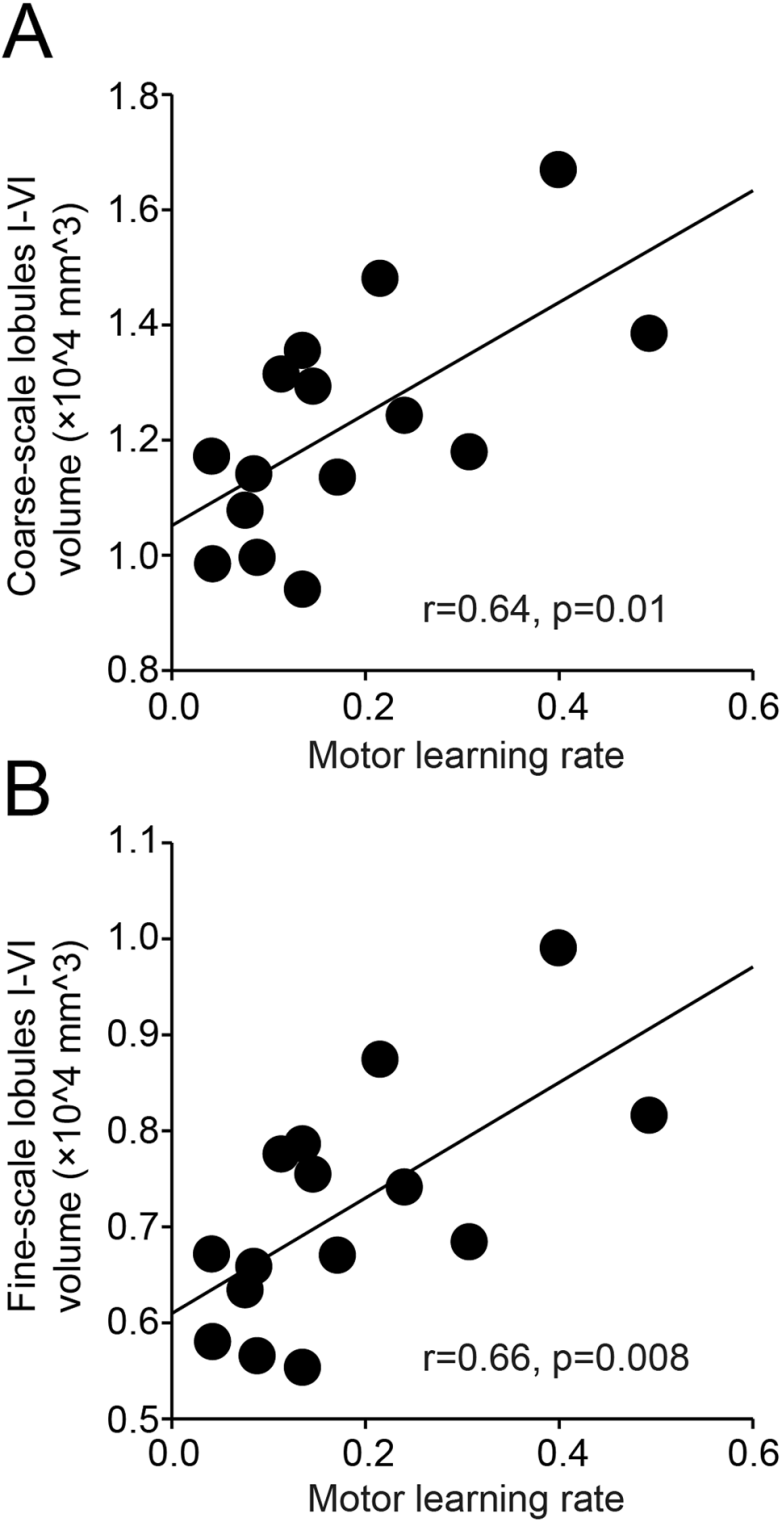
Cerebellar changes and motor learning rates. (**A**, **B**) Graphs show a correlation between motor learning rates and the volume of coarse- and fine-scale sensorimotor cerebellar lobules I–VI in SCI subjects (SCI = 15). Note that SCI subjects with slower motor adaptation learning rates were those with smaller volume of coarse- and fine-scale sensorimotor cerebellar lobules I–VI. Error bars indicate SDs. *p < 0.05.

We found a correlation between time post-injury and the volume of the sensorimotor cerebellum (Fig. 5A) in SCI subjects. Here, SCI subjects with increasing time post-injury were those with smaller volume in the coarse- (r = 0.65, p = 0.005; Fig. 5B) and fine- (r = 0.63, p = 0.006; Fig. 5C) scale sensorimotor cerebellar lobules I–VI. We also found a correlation between motor learning rates and the volume of coarse- and fine-scale sensorimotor cerebellar lobules I–VI in SCI subjects. Note that SCI subjects with slower motor adaptation learning rates were those with smaller volume of coarse- (r = 0.64, p = 0.01; Fig. 6A) and fine-scale (r = 0.66, p = 0.008; Fig. 6B) sensorimotor cerebellar lobules I–VI. Since a decline in cerebellar volume and adaptation learning associates with aging^37–39^, we performed additional analysis to assess the contribution of aging to our effects. We found no correlation between age and the volume of coarse- (r = 0.30, p = 0.24) and fine-scale (r = 0.29, p = 0.25) sensorimotor cerebellar lobules I–VI in SCI subjects. Similarly, aging was not correlated with motor learning rates in SCI subjects (r = 0.06, p = 0.83). Furthermore, we conducted a multiple regression analysis to examine associations between the learning rate and cerebellar volume, age, years post-injury, handedness, upper extremity position sense, and muscle strength. We found that some of these variables predicted learning rate (F_(6, 9)_ = 15.15, p < 0.001). Specifically, cerebellar volume (p = 0.001) and years post-injury (p = 0.03), but not age (p = 0.6), handedness (p = 0.8), upper extremity position sense (p = 0.2), and muscle strength (p = 0.3), were predictors of the learning rate.

## Discussion

Two main novel findings arise from our study. First, we found that humans with chronic incomplete cervical SCI have a reduced ability to adapt their movements during a visuo-motor rotation task involving reaching movements compared with uninjured controls. Specifically, individuals with SCI showed a decreased ability to learn from movement errors compared with control subjects. Second, structural imaging data showed that sensorimotor regions in the cerebellum were reduced in size in SCI compared with control subjects, with cerebellar atrophy increasing with time post-injury. The degree of spared tissue in the cerebellum was positively correlated with motor adaptation learning rates, suggesting that participants with lesser atrophy showed higher learning rates. We hypothesized that abnormalities in spinocerebellar structures contribute to motor adaptation deficits in humans with chronic incomplete cervical SCI.

### Sensorimotor adaptation learning after SCI

Sensorimotor adaptation learning has been widely studied in humans by distorting the relationship between hand movement and visual feedback—a visuo-motor rotation task^13^. This learning results in a practice-dependent reduction of errors detected from sensory feedback and plays an important role in skilled motor behaviors and rehabilitation of patients with motor disorders^1,2^. Here, we demonstrate for the first time that humans with chronic SCI showed slower learning rates and smaller after-effects when adapting to a visuo-motor rotation task compared with control subjects. These results are consistent with evidence showing that tasks requiring visuo-motor integration are difficult for individuals with SCI^8–10^. This also agrees with studies showing that after SCI there is a decreased ability to form internal body representations^11,12^. Reaching movements in our task required more use of elbow flexor or extensor muscles depending on the direction of the movement tested^40^. As in previous studies^41,42^, we found that individuals with SCI had stronger biceps compared with triceps brachii voluntary output^43^. A separate analysis of trials where reaching required more elbow extension or flexion revealed similar learning rates, suggesting that it is less likely that this factor contributed to our results in SCI participants. Evidence also showed that distinct control mechanisms contribute to the dominant and non-dominant arm for adaptation learning^33,34^. In this study, we tested the dominant arm in controls and the “more affected arm” in SCI subjects. Our results showed that the handedness of SCI subjects is affected after the injury, in which 13/22 of SCI subjects switched the dominant arm to the “more affected arm” after the injury. These SCI subjects also showed lower learning rate and mean learning index compared with controls during the 30° visuo-motor rotation, suggesting that it is less likely that this factor influenced our results.

### Cerebellar contribution to sensorimotor adaptation learning after SCI

An important question is why humans with SCI showed deficits in learning a visuo-motor rotation task compared with control subjects. We favor the hypothesis that the presence of cerebellar atrophy contributed to our findings. Atrophy was found in sensorimotor regions of the cerebellum in SCI compared with control subjects. This agrees with studies showing cerebellar atrophy^31,32^ and altered cerebellar activation patterns during voluntary activity^44^ in humans with SCI. Note that we examined regions of the cerebellum known to be important for sensorimotor control and reaching adaptation tasks. Our coarse-scale analysis focused on a cerebellar region that exhibits large resting state connectivity with the sensorimotor network of the cerebral cortex whereas the fine-scale analysis focused on the hand and foot representation^45,46^. Neuroimaging studies in humans showed that within the cerebellum sensorimotor lobules I–VI largely contribute to the learning of a visuo-motor rotation task during reaching movements^6,17,18^. We found pronounced atrophy on lobules I–VI in SCI participants compared with control subjects when using both the coarse- and fine- scale analysis. Thus, it is possible that the change in cerebellar size is a main factor contributing to our results. This is supported by the positive correlation found between the degree of spared tissue in the cerebellum and learning rates, indicating that individuals with lesser atrophy showed higher learning rates. Indeed, patients with cerebellar degeneration showed that atrophy in the sensorimotor cerebellum correlates with visuo-motor rotation adaptation learning rates^5,6,47^. Purkinje cells encode error signals during sensorimotor adaptation learning^25,48^ and the number^26^ and synaptic contacts^19^ of Purkinje cells decreases after SCI. Lower Purkinje cell activity may result in a reduced cerebellar size and subsequently lead to impairments in learning from errors. Spinocerebellar neurons conveying proprioceptive and cutaneous information from Golgi tendon organs and muscle spindles terminate in cerebellar lobules I–VI^20,49,50^. Most injuries to the spinal cord in humans are associated with high-energy fracture-dislocations and burst fractures that affect several regions of the spinal cord^51^. Because the spinocerebellar tract occupies a large portion of the white matter spanning across dorsal, lateral, and ventral areas^52^, it is likely that this tract is affected by the injury. Indeed, our SCI participants showed deficits in upper extremity position sense compared with control subjects, agreeing with an impairment on spinocerebellar neurons^53^.

It is important to note that the magnitude of cerebellar atrophy correlated with the time post SCI, showing that participants with longer time post-injury showed larger atrophy. This is consistent with results showing that cortical white and gray matter atrophy increases with the time after SCI^31,32^. Specifically, participants with injuries for more years showed larger cerebellar atrophy than individuals with injures for fewer years. This agrees with recent results showing that differences exist in proprioceptive function in humans who had SCI for more and less than 10 years^54^. It is also possible that aging contributed to our results. Evidence showed that the volume of the cerebellum decreases with age in control subjects and note that older individuals have different learning rates compared with young individuals^37–39,55^. However, our results showed no correlation between age and the volume of coarse- and fine-scale sensorimotor cerebellar lobules I–VI in SCI subjects, suggesting that it is less likely that this factor contributed to our findings. Proprioceptive signals are important for sensorimotor adaptation learning^29,30,56^ and often altered after SCI^7^. However, deafferented subjects can learn to adapt to movement errors^57–59^ and our results showed that impairments in proprioception were not correlated with deficits in motor learning in SCI subjects, suggesting that other factors are also important to consider. For example, sensorimotor adaptation learning involves contributions from other brain regions such as the parietal cortex^17^ and functional connectivity between cortical regions decreases after SCI^60,61^, including parietal areas^62^. It is also possible that damage to spinal cord networks contributed to our findings. The spinal cord plays an important role in human motor learning^63^, including learning of a visuo-motor task^64^. Recent neuroimaging studies in humans showed that learning related BOLD signal changes in the spinal cord is functionally synchronize with changes in brain regions, including the cerebellum^65^, making it difficult to separate these influences. Although we cannot completely exclude the possibility that other factors also affected our results, our findings support the view that the reduced ability to learn from movement errors during reaching movements in humans with SCI involves abnormalities in the spinocerebellar structures.

### Functional considerations

Errors signals play an important role in helping the motor system to correct movements^66^. On one side, it is possible that the differences that we found in arm deceleration between patient and control subjects may reflect that patients are more likely to learn via on-line motor corrections (as no time-constraint was given) rather than just learning via sensory prediction errors, consistent with previous results^67^. On the other side, the reduced ability to learn from sensory prediction errors might have important implications for rehabilitation. For example, during practice, subsequent movements that depend on a previous position estimate could be more accurate by using these predictions. In other words, planning the next movement can benefit from the efference copy of the preceding movement. Since sensory input is often altered after SCI^7^, an advantage of making sensory predictions is that the brain does not have to wait for sensory input to act. For example, studies using body-machine interfaces found that humans with SCI improve their limb function by learning a predictive map between their residual motion and a low dimensional control space^68,69^. If cerebellar atrophy limits the ability to adapt movements to new demands, some strategies might be helpful to consider. Non-invasive stimulation of the cerebellum can influence the ability to learn from movement errors^70–72^ and might represent a strategy to consider in future studies aiming to enhance visuo-motor interactions after SCI.

## Methods

### Subjects

Twenty-two individuals with SCI (mean age 46.9 ± 14.5 years, 4 female, range 20 to 70 years; Table 1) and 22 age-matched controls (mean age 41.3 ± 13.2 years, six female, range 20 to 70 years; p = 0.3) participated in the study. All subjects gave informed consent to the experimental procedures, which were approved by the local ethics committees at the University of Miami. The study was performed in accordance with the Declaration of Helsinki. Individuals with SCI had a chronic (≥ 1 year) cervical injury (C2–C6), an intact or impaired, but not absent, innervation in dermatomes C6 using the International Standards for Neurological Classification of Spinal Cord Injury (ISNCSCI) sensory scores and residual hand and arm motor function. All SCI subjects were able to control arm movements without compensatory trunk movements. Four out of 22 SCI subjects were categorized by the American Spinal Cord Injuries Association Impairment Scale (AIS) as AIS A (complete injury) due to the lack of sacral sparing^73^, despite being able to exert voluntary force in arm muscles. Seventeen subjects were classified as incomplete AIS B, C, and D (Table 1). As previously shown^74,75^, we determined the more and less affected arm in SCI participants by averaging the maximum isometric voluntary contraction (MVC) quantified using background EMG activity in all hand and arm muscles tested, including the first dorsal interosseous, abductor pollicis brevis, extensor digitorum communis, flexor digitorum superficialis, biceps brachii, triceps brachii, anterior deltoid, and posterior deltoid (SCI less affected arm = 0.32 ± 0.24 mV, SCI more affected arm = 0.24 ± 0.19 mV; p = 0.001). The arm detected as the “more affected arm” by using MVCs was also confirmed by the subjects’ own estimation of their “more affected arm” in 20/22 SCI subjects. In control subjects, we tested the dominant arm and in participants with SCI, we tested the “more affected arm”. SCI largely damages both sides of the spinal cord resulting in asymmetrical deficits in sensory and motor function^54,74^. Thus, the handedness of SCI participants is affected by the injury^76^. Thirteen SCI subjects were right-handed before the injury and this arm corresponded to the “more affected arm” after the injury. Additional analysis was conducted on these individuals to assess the effect of handedness on learning outcomes. Note that all SCI participants included in the study were able to perform reaching movements to all targets with the “more affected arm” without limitations. Fifteen SCI and control subjects participated in all experiments described below. We tested a lesser number of SCI subjects for the MRI portion of the study because one of the participants had difficulties lying flat on the scanning table, 2 of the participants had problems with bullet fragments or hardware compatibility, and 4 participants could not return for other experiments.

**Table 1:** Spinal cord injury participants.

SCI subject	Age (years)	Gender	Level	ASIA score	Aetiology	Time post injury (years)
1	53	F	C5	C	T	14
2	51	F	C2	A	T	10
3	33	M	C2	C	T	9
4	58	F	C4	D	T	18
5	36	M	C5	C	T	4
6	69	M	C2	C	T	5
7	31	M	C5	A	T	12
8	47	M	C5	D	T	4
9	61	M	C5	C	T	17
10	66	M	C4	D	T	6
11	66	M	C5	A	NT	6
12	59	M	C5	C	T	9
13	39	M	C5	D	T	12
14	60	M	C4	D	NT	5
15	36	M	C6	C	T	17
16	51	M	C2	D	T	5
17	23	M	C5	A	T	4
18	60	M	C5	D	T	16
19	44	M	C4	C	T	5
20	21	M	C2	C	T	5
21	33	F	C4	C	T	14
22	36	M	C5	C	T	4

*M* male, *F* female, *ASIA* American Spinal Injury Association Impairment Scale, T traumatic, *NT* nontraumatic.

### Apparatus

Participants were asked to make reaching movements to targets in the horizontal plane while seated with the more affected arm in SCI and the right arm in control subjects was supported by a robotic exoskeleton. The robotic exoskeleton (KINARM, BKIN Technologies) can apply independent mechanical loads to the shoulder and/or elbow joints^77,78^. The trunk was securely strapped to the chair with a harness to minimize compensatory trunk movements. The linkages of the exoskeleton were adjusted to custom-fit to each subject according to their limb length and geometry. The chair was moved to bring the arms under a horizontal display. Visual targets and hand-aligned feedback were projected into the plane of the participant’s hand via a virtual reality display and semi-silvered mirror. Direct vision of the subject’s hand and arm was blocked with a physical barrier and a cursor representing their index fingertip (0.5 cm diameter) was provided to guide the reaching movements (white circle; Fig. 1B). Computer algorithms used for data processing and analysis were written in MATLAB (The Mathworks Inc., Natick, MA).

### Experimental paradigm

Individuals were asked to control a white cursor while performing reaching movements from a start circle to one of four targets. The distance between the start circle and the four targets was 10 cm. Note that 10 cm is a length that fitted all our subjects according to their limb length and geometry and it is widely used during visuomotor adaptation tasks^79–81^. Each of the four targets was randomly presented within every cycle and the same target did not occur twice in each cycle. For each trial, subjects first moved the finger (cursor) to the start circle, and then they reached out towards the target in a rapid and straight manner when the target appeared. Continuous visual feedback of the finger (cursor) trajectory was provided throughout the entire reaching movement in each trial. Movements were required to have a reaction time of < 800 ms and a movement time within 600–1200 ms. The target disappeared 800 ms after its appearance. The color of the target turned red or blue if the movement was too fast or too slow, respectively. The target color turned green when the performance met the requirement. The experiment consisted of three sessions: baseline (80 trials), adaptation (100 trials), and de-adaptation (100 trials). During the baseline session, subjects were familiarized with the reaching movement without external perturbations. During the adaptation session, subjects adapted to a visual display that was rotated 30° counterclockwise relative to the actual movement (Fig. 1C). 30° of visual perturbation is less likely influenced by explicit cognitive strategies^82^ and is largely used during visuomotor adaptation tasks^79–81^.

During the de-adaptation session, subjects performed reaching movements without the visual rotation perturbation. We provided 1 min of rest between sessions and additional rest as needed to avoid fatigue. Reaching movements to the 4 targets tested required more use of elbow flexors or elbow extensors depending on the direction of the movement^40^. Consistent with previous findings^41,42^, we found that MVC (measured as the highest mean rectified EMG activity found in 1 s during the MVC burst) was higher in biceps (0.59 ± 0.30 mV) compared with triceps (0.24 ± 0.11 mV; p = 0.001; Fig. 3B) brachii in SCI participants. Therefore, we completed a separate analysis on trials when reaching to the target required elbow extension (Elbow Ext = target positioned in the midline in front of the start circle) or elbow flexion (Elbow Flex = target positioned in the midline behind the start circle).

### Kinematic recordings

The 2-D position data of the hand, elbow, and shoulder were sampled at 1000 Hz and digitally low-pass filtered at 15 Hz. To assess task performance, we calculated three variables. (1) Direction error: the angular difference between a vector from the start circle to the target and another vector from the hand position at movement start to that at peak arm velocity. (2) Learning rate: obtained by fitting a single decaying exponential function to the time series with the assumption that learning is monotonic^83–85^: Errors (n) = A × exp (− R × n) + B. R was the individual learning rate, A and B were two constants, and n was the trial index. (3) Learning index: the amount of learning during the adaptation session for each subject based on the following equation:

$$Learning \, index=\frac{\left|{MD}_{adaptation \, trials}-{MD}_{baseline \, trials}\right|}{\left|Rotation \, size-{MD}_{baseline \, trials}\right|}$$

where MD is movement direction, and the rotation size is 30°. The learning index computation takes into account baseline trials in adaptation trials, whereas direction error and learning rate only capture motor performance in adaptation trials. The learning index ranged from 0 to 1 with 1 indicating maximal learning and 0 indicating no learning. The denominator is the expected maximal magnitude of changes in movement directions in adaptation trials. The numerator is the actual magnitude of changes in adaptation trials. If subjects adapt to the visuomotor rotation completely, the direction of the arm trajectory should be 30° away from the target, which makes the numerator equals the denominator (learning index = 100%). If the subject’s performance is no different from the baseline, the numerator is zero, so the learning index = 0%. We also assessed other kinematic features of the movement. Reaction time: the time between the presentation of a target and the onset of a reaching movement. Arm acceleration: the time between movement onset and peak arm velocity. Arm deceleration: the time between peak arm velocity and movement offset.

### Electromyographic (EMG) recordings

EMG was recorded from the biceps and triceps brachii muscles through bipolar electrodes (Ag–AgCl; 10-mm diameter). Subjects performed 2–3 MVCs for 3–5 s with the muscles tested, separated by 1 min of rest. EMG signals were amplified, filtered (bandwidth 30–2000 Hz), and sampled at 5 kHz for off-line analysis.

### Upper extremity position sense and clinical assessment

The assessment of upper extremity position sense was conducted for both arms using the arm-position matching task^35^. To assess position sense for the “more affected” arm, the “more affected” arm was passively moved to 9 targets presented randomly while subjects were asked to actively mirror-match the position with the “less affected” arm. Note that the arm that the participant was required to move was the same arm that the participant was required to move to reach towards the targets during the adaptation experiment. Targets were separated by 10 cm and the direct vision of the subject’s hand and arm was blocked with a physical barrier. Subjects were given as much time as needed to complete each trial. Trials were started when subjects verbally notified the examiner that they completed the preceding trial. During passive movements, the KINARM exoskeleton moved the subject’s arm straight from one target to another without going back to the center position. Fifty-four trials (6 repetitions per target) were completed in total. Position accuracy was assessed by measuring the mean error between the active and passive hands for each target-matching position across a series of trials. Clinical assessment of arm/hand motor function was conducted by using the Jebsen Taylor Test^36^. SCI subjects performed the following subtests: card turning, picking up small common objects, simulated feeding, moving light objects (i.e. empty cans), and moving heavy objects (i.e., 11b cans). Each task was performed on a wooden board and it was timed with a stopwatch.

### MRI

Magnetic resonance images of SCI and control subjects were acquired with a 3-T Siemens Sonata Scanner (Siemens, Erlangen, Germany). Structural brain imaging was obtained using a T_1_-weighted, magnetization-prepared gradient-echo sequence. Three subjects were excluded from further analysis due to poor image quality because of severe motion artifact. The SUIT toolbox was implemented to isolate the cerebellum from the surrounding tissue and the resulting segmented images were then normalized to the SUIT space^45^, which produced a deformation matrix between the standardized template and the individual’s cerebellum. The sensorimotor cerebellum was separated from other cerebellar regions by using the templates 7-network (coarse-scale) and 17-network (fine-scale) cerebellar parcellations^46^. Note that the coarse-scale sensorimotor region exhibits the largest resting state connectivity to the motor and somatosensory cortices, whereas the fine-scale sensorimotor region involves the hand and foot representations^46,47^. Sensorimotor adaptation learning particularly relies on anterior and posterior cerebellar lobules I–VI^4,17^. Therefore, we also identified individual cerebellar lobules I–VI and the sensorimotor topography of cerebellar lobules I–VI. The volume of the sensorimotor cerebellum and the individual cerebellar lobules of each subject were measured by multiplying separated regional volumes by the density of the voxels in those regions.

### Data analysis and statistics

Normal distribution was tested by the Shapiro–Wilk’s test and homogeneity of variances by the Levene’s test of equality and Mauchly’s test of sphericity. When normal distribution could not be assumed, data were log transformed. When sphericity could not be assumed, the Greenhouse–Geisser correction statistic was used. Repeated measures ANOVAs were performed to determine the effect of GROUP (controls and SCI) and CYCLE [initial bias (cycle 1), early learning (mean of cycle 2–6), and asymptote learning (mean of cycle 21–25)] on direction error and learning index. The same analysis was used to determine the effect of TARGET (Elbow Flex and Elbow Flex) and CYCLE on direction error and learning index. Independent t-tests were used to compare the learning rate, direction error from the first cycle during de-adaptation, and cerebellar volume between SCI and control subjects. Tukey post hoc analysis was used to test for significant comparisons. Pearson correlation analysis was used to assess the relationship between cerebellar volume and years post-injury and between cerebellar volume and motor learning rate corrected for multiple comparisons using a Bonferroni correction. Significance was set at p < 0.05. Group data are presented as the mean ± SD in the text.
